# Life stage and proximity to roads shape the skin microbiota of eastern newts (*Notophthalmus viridescens*)

**DOI:** 10.1111/1462-2920.15986

**Published:** 2022-04-29

**Authors:** Vanessa P. Wuerthner, Jessica Hua, Obed Hernández‐Gómez

**Affiliations:** ^1^ Department of Biological Sciences Binghamton University Binghamton NY; ^2^ Department of Forest and Wildlife Ecology University of Wisconsin‐Madison Madison WI; ^3^ Department of Environmental Sciences, Policy, and Management University of California‐Berkeley Berkeley CA; ^4^ Department of Natural Sciences and Mathematics Dominican University of California San Rafael CA

## Abstract

Host‐associated microbiomes play an essential role in the health of organisms, including immune system activation, metabolism and energy uptake. It is well established that microbial communities differ depending on the life stage and natural history of the organism. However, the effects of life stage and natural history on microbial communities may also be influenced by human activities. We investigated the effects of amphibian life stage (terrestrial eft vs. aquatic adult) and proximity to roadways on newt skin bacterial communities. We found that the eft and adult life stages differed in bacterial community composition; however, the effects of roads on community composition were more evident in the terrestrial eft stage compared to the aquatic adult stage. Terrestrial efts sampled close to roads possessed richer communities than those living further away from the influence of roads. When accounting for amplicon sequence variants with predicted antifungal capabilities, in the adult life stage, we observed a decrease in anti‐fungal bacteria with distance to roads. In contrast, in the eft stage, we found an increase in anti‐fungal bacteria with distance to roads. Our results highlight the need to consider the effects of human activities when evaluating how host‐associated microbiomes differ across life stages of wildlife.

## Introduction

Host‐associated microbiomes play an essential role in the health of organisms, including immune system activation, metabolism and energy uptake (Petersen and Round, 2014). It has been well‐established that microbial communities may differ depending on the life stage and natural history of the organism. Dramatic morphological and habitat shifts through development (i.e. metamorphosis) alter the microbial diversity and community composition of both vertebrate and invertebrate species (Wang *et al*., 2011; Kohl *et al*., 2013; Kueneman *et al*., 2014; Jimenez and Sommer, 2017). For example, the microbial community structure in the gut of the mosquito, *Anopheles gambiae*, differed significantly between immature stages (i.e. larvae and pupae) and adults (Wang *et al*., 2011). Similarly, in amphibians, fire‐bellied toads (*Bombina orientalis*) experienced a major reorganization of the skin microbial communities during metamorphosis (Bataille *et al*., 2018). Both mosquitos and many amphibian species utilize both aquatic and terrestrial habitats, depending on the stage of development. These differences in environment play a major role in structuring skin and gut microbial communities (Kueneman *et al*., 2014; Walke *et al*., 2014; Avena *et al*., 2016; Jani and Briggs, 2018).

As our understanding of microbiomes continues to grow, it is also well documented that the environment plays a major role in structuring host‐associated microbial communities (Kueneman *et al*., 2014; Walke *et al*., 2014; Avena *et al*., 2016; Jani and Briggs, 2018). While the effects of environmental variation on host‐associated microbiomes have been studied extensively in humans, ruminants and model organisms, this line of study has not been as deeply investigated in wildlife. For example, habitat degradation (i.e. fragmentation and deforestation) can alter both skin and gut microbial communities of organisms by altering natural sources of microbial symbionts in the environment (Barelli *et al*., 2015; Becker *et al*., 2017; Hughey *et al*., 2017; Jimenez *et al*., 2020; Barnes *et al*., 2021; Neely *et al*., 2021). Similarly, exposure to human‐made chemicals in the environment has been shown to influence associations between hosts and microbial symbionts, in many cases resulting in negative effects to host health (Redford *et al*., 2012). Given the association between host‐associated microbiomes and health, characterizing natural variation in wildlife microbiomes, and identifying sources of dysbiosis are key to fully understanding the effects of environmental change on wildlife. Importantly, because habitat types may impose different selective pressures on hosts and their microbial commensals, organisms that utilize different habitats across various life stages may be differentially impacted by human activities. This underscores the importance of evaluating the interactive effects of life stage and environment on microbial symbionts in natural populations.

With over 4 million miles of roads, it is estimated that nearly 15%–20% of the United States is ecologically impacted by roads (Forman and Alexander, 1998; Forman, 2000). While the direct effects of roads (i.e. mortalities) on wildlife have been well documented, the indirect effects roads have on natural populations are less understood (Acevedo‐Whitehouse and Duffus, 2009). For example, roads may modify habitat connectivity, amphibian assemblages and genetic diversity (Glista *et al*., 2008), all of which can be associated with variation in microbial communities (Becker *et al*., 2017). Additionally, roads are often associated with an increase in exposure to chemical contaminants (e.g. agrochemicals, heavy metals and road salts), which also have been documented to alter both wildlife hosts and their microbial communities (Narrowe *et al*., 2015; McCoy and Peralta, 2018; Hernández‐Gómez *et al*., 2020). For instance, road salt (NaCl), a common chemical contaminant used on roadways, has been shown to decrease microbial abundance and growth in aquatic plants (O'Brien *et al*., 2020). Despite the potential for roadways to influence host‐associated microbiotas, the effect that proximity to roads may have on an organism's microbial symbionts and its ultimate indirect effect on health and survival are relatively less understood.

Amphibians serve as a good model system for monitoring skin microbial changes between life stages and in response to habitat change. First, most amphibians go through metamorphosis, where microbial communities have been shown to change substantially due to anatomical and physiological changes in the integumentary and immune systems (Rollins‐Smith, 1998; Kueneman *et al*., 2014). Understanding the impacts roads have on amphibian populations is important given that habitat modification and degradation is one of the leading causes of global amphibian declines (Hayes *et al*., 2010), with road mortality being a significant contributor (Gibbs and Shriver, 2005; Glista *et al*., 2008; Elzanowski *et al*., 2009; Beebee, 2013). Additionally, amphibians that use habitats near roadways are often exposed to greater amounts of pollutants, including heavy metals and de‐icing agents (Norrström and Jacks, 1998; Sanzo and Hecnar, 2006). Notably, microbiota are often the first taxa to respond to pollutants (Lew *et al*., 2009; McCoy and Peralta, 2018), but those effects are dependent on the contaminant. For example, short‐term exposure to coal combustion waste had little impact on the skin microbial communities of spring peepers (*Pseudacris crucifer*; Hughey *et al*., 2016), whereas exposure to an agriculture antimicrobial induced a change in the skin microbial composition in Northern leopard frogs (*Lithobates pipiens*; Hernández‐Gómez *et al*., 2020). This is important because the skin of amphibians, and the microbes that reside there, are highly sensitive to environmental changes, which may have implications for the relationship between skin microbial symbionts and amphibian susceptibility to disease [i.e. *Batrachochytrium dendrobatidis* (Bd); McCoy and Peralta, 2018; Hernández‐Gómez *et al*., 2020]. In addition, several bacterial isolates collected from the skin of amphibians can produce metabolites that inhibit the growth of Bd (Woodhams *et al*., 2015; Woodhams *et al*., 2018). Given the link between changes to the skin microbiota and amphibian health, plus the potential role of bacterial symbionts in disease dynamics, the interference of roads through habitat degradation and introduction of pollutants may negatively impact amphibian populations across multiple life stages.

Using the eastern newt (*Notophthalmus viridescens*) as our amphibian model, we investigated the effects of amphibian life stage (i.e. terrestrial eft vs. aquatic adult) and proximity to roadways on newt skin bacterial communities. Specifically, we examined whether changes in microbial diversity and composition differed between eft and adult newt populations across habitats that were close and far from roads. To evaluate the effect of life stage and road proximity on the protective phenotype of the skin microbiome, we identified bacteria with potential Bd‐inhibiting properties using a DNA sequence database. We determined whether changes in Bd‐inhibitory microbial diversity and relative abundance differed between eft and adult newt populations across habitats that were close and far from roadways. We predicted that: (i) the terrestrial eft stage would exhibit higher microbial diversity than the aquatic adult newt stage and community composition between the two life stages would differ, as shown in previous research (Sabino‐Pinto *et al*., 2017); (ii) populations located in close proximity to roads would have lower bacterial diversity compared to those located far from roads (O'Brien *et al*., 2020), while also exhibiting distinct bacterial communities; (iii) the effect of roadways on the skin microbiota diversity and abundance of Bd‐inhibitory microbiota would depend on life stage, because terrestrial juvenile and aquatic adult life stages utilize different habitats.

## Results

We located four eastern newt populations and collected 10 terrestrial juvenile (eft) and 10 aquatic adult eastern newts from each population on 24–25 July 2019. Evidence suggests that edge effects from roads can extend up to 240 m into natural habitats (Franklin and Forman, 1987; Chen *et al*., 1995; Forman and Alexander, 1998), therefore, we chose two populations located far (>250 m) from roads [Aqua‐Terra (AT) and Florence Shelly Preserve (FS)], and two located close (<250 m) to roads [Nature Preserve (NP) and Nuthatch Hollow (NH); Table 1]. We performed Illumina sequencing on the 16S rRNA V4 amplicons produced from the DNA extracted from skin swabs. We processed 1 547 723 DNA sequence reads using QIIME2 to produce 11 771 amplicon sequence variants (ASVs) following contaminant and non‐bacteria taxonomy removal. We also generated a Bd‐inhibitory ASV table with only ASV's whose representative sequence matched the 16S rRNA sequences of Bd‐inhibiting bacteria at a 97% identity match or higher. We deposited the raw 16S rRNA V4 amplicon sequencing data into the NCBI Sequence Read Archive (project Accession Number: PRJNA783030).

**Table 1:** The population abbreviations, coordinates, and distances to roads (m) for each of the eastern newt populations used in this study.

Population	Latitude (N)	Longitude (W)	Distance to road (m)
NP	42°05′01.6″	75°58′22.5″	84.96
NH	42°04′53.4″	75°59′11.1″	231.83
AT	42°01′56.6″	75°56′30.7″	428.61
FS	41°53′01.6″	75°30′24.7″	952.78

All values are derived from Google Earth Pro.

### Effects of life stage and roads on bacterial diversity and composition

**Fig. 1:**
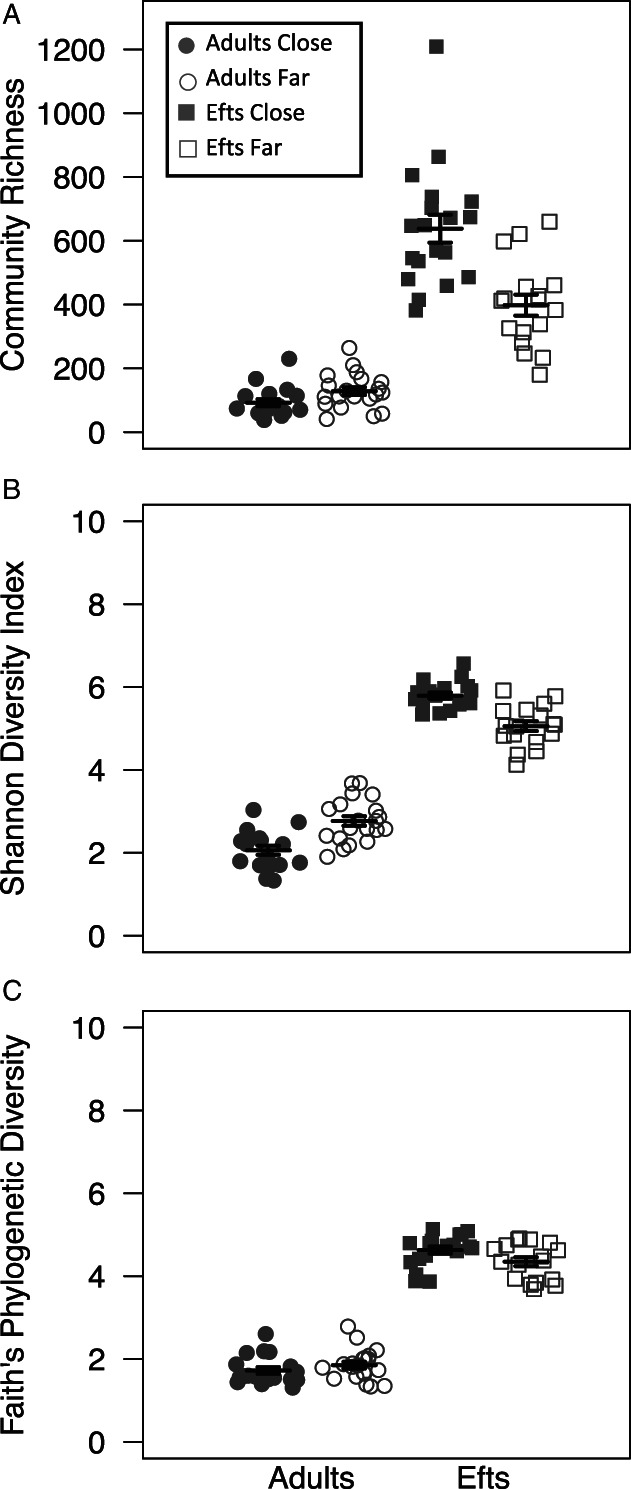
Alpha diversity dot plots with mean (long horizontal line) and standard errors (short horizontal line). Alpha diversity measures presented for adult and eft eastern newts include community richness (A), Shannon Diversity index (B) and Phylogenetic Diversity (C). Each point represents the bacterial skin community of an individual sample; point colour indicates proximity to roads (grey is close to roads and white is far from roads) and shape identity indicates newt stage (circles are adult newts and squares are efts).

We also evaluated differences in newt skin microbial diversity and composition between two eastern newt life stages (i.e. eft vs. adult) and four populations that varied in their distance to roads. The backwards reduced fixed‐effect procedure during linear mixed model selection kept the life stage and road proximity variables in the models fitting community richness, phylogenetic diversity and Shannon diversity. Overall, efts had significantly higher community richness (*F*_1,70.03_ = 355.28, *p* < 0.001), phylogenetic diversity (*F*_1,68.02_ = 225.02, *p* < 0.001) and Shannon diversity (*F*_1,68.01_ = 982.78, *p* < 0.001) compared to adult newts (Fig. 1). Proximity to roads alone had no significant effect on newt community richness (*F*_1,4.01_ = 0.23, *p* = 0.656), phylogenetic diversity (*F*_1,2.00_ = 1.11, *p* = 0.402), or Shannon diversity (*F*_1,2.00_ = 0.0015, *p* = 0.972); however, the interaction between life stage and proximity to roads was significantly associated with community richness (*F*_1,70.02_ = 24.28, *p* < 0.001), phylogenetic diversity (*F*_1,68.02_ = 29.47, *p* < 0.001) and Shannon diversity (*F*_1,68.01_ = 56.37, *p* < 0.001). Across all alpha diversity metrics, efts living close to roads had higher alpha diversities than those far from roads, whereas the opposite pattern was true in adult newts (i.e. higher alpha diversities among individuals living further away from roads; Fig. 1).

**Fig. 2:**
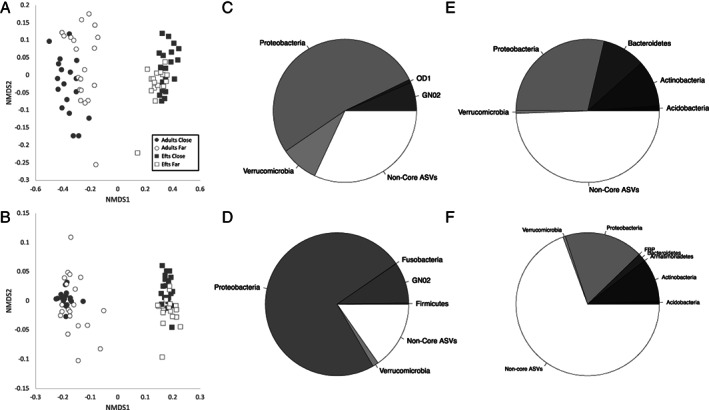
Compositional diversity of adult and terrestrial efts living in habitats close and far from roads. NMDS plots of unweighted UniFrac (A) and weighted UniFrac (B) distance matrices from adult and eft eastern newts. Each point represents the bacterial skin community of an individual sample; point colour indicates proximity to roads (grey is close to roads and white is far from roads) and shape identity indicates newt stage (circles are adult newts and squares are efts). Unweighted UniFrac NMDS stress: 12.77%; weighted UniFrac NMDS stress: 12.79%. Phylum composition of the core microbiomes (i.e. ASVs present in at least 50% of individuals) for adult newts living far from roads (C), adult newts living close to roads (D), efts living far from roads (E) and efts living close to roads (F).

We detected differences in community composition between life stage, distance to roads and their interaction using both beta diversity metrics (i.e. unweighted and weighted UniFrac; Table 2). The PERMANOVA test demonstrated that life stage explained most of the variation in our model (unweighted UniFrac *R* ^2^ = 0.25, *p* = 0.001; weighted UniFrac *R* ^2^ = 0.65, *p* = 0.001), while proximity to roads and the interaction between roads and life stage explained less than 5% of the variation across both unweighted (*R* ^2^ = 0.03, *p* = 0.001) and weighted UniFrac (*R* ^2^ = 0.02, *p* = 0.007) comparisons (Table 2). To test how life stage or proximity to roads influences the heterogeneity of the newt skin microbiomes, we calculated and evaluated beta diversity heterogeneity (i.e. distance to group centroid) using linear mixed models. Unweighted UniFrac dispersion was significantly associated with both proximity to roads (*F*_1,74_ = 7.55, *p* = 0.008) and life stage (*F*_1,70.24_ = 33.07, *p* < 0.001); whereas we did not observe any differences in weighted UniFrac dispersion among the groups of newts compared. Adult newts (mean ± standard error: 0.56 ± 0.0044) possessed slightly higher unweighted UniFrac dispersion values than efts (0.52 ± 0.0030), and newts living close to roads (0.55 ± 0.0054) possessed higher unweighted UniFrac dispersion values than those living further away (0.53 ± 0.0044). We visualized clustering of samples between the different life stages and distance to roads using NMDS plots generated using unweighted and weighted UniFrac metrics (Fig. 2A and B). The core microbiomes, defined here as the ASVs present in 50% of the population, of adult newts were dominated by Proteobacteria (>50% relative abundance), regardless of whether they were collected close or far from roads (Fig. 2C and D). In contrast, the core microbiomes of efts were more evenly distributed between Proteobacteria, Bacteroidetes and Actinobacteria (Fig. 2E and F).

**Table 2:** Two‐way PERMANOVA test among microbial communities across stage and proximity to roads from four eastern newt populations.

Groups	Unweighted UniFrac	Weighted UniFrac
Stage	*R* ^2^ = 0.25	*R* ^2^ = 0.65
	Pseudo *F*_1,73_ = 25.24	Pseudo *F*_1,73_ = 153.75
	*p* = 0.001	*p* = 0.001
Proximity to roads	*R* ^2^ = 0.03	*R* ^2^ = 0.02
	Pseudo *F*_1,73_ = 3.16	Pseudo *F*_1,73_ = 5.90
	*p* = 0.001	*p* = 0.007
Interaction	*R* ^2^ = 0.03	*R* ^2^ = 0.03
	Pseudo *F*_1,73_ = 3.08	Pseudo *F*_1,73_ = 6.52
	*p* = 0.004	*p* = 0.004

Pseudo *F*‐statistics and *p*‐values are presented for each comparison performed on weighted and unweighted UniFrac distances.

The indicator species analysis identified 128 ASVs associated with adults and 769 associated with efts (Table S1). Additionally, 133 ASVs were associated with proximity to roads and 69 were associated with far distances to roads (Table S1). Most of the ASVs associated with all sample groups belong to the phyla Proteobacteria, Bacteroidetes, Actinobacteria or Acidobacteria. While most ASVs had a relative abundance under 1%, a single Bacteroidetes ASV (*Pedobacter* sp.) had a relative abundance of 5.90% in efts captured far from roads. This same ASV was also found in low relative abundances in efts close to roads (0.37%) and adults close to roads (0.0009%) and was not found in adults far from roads (Table S1). Given that most of the ASVs that differed between life stages and road proximity are found at low relative abundances on the skin of the newts, we can infer that mostly the *Pedobacter* ASV and rare ASV turnover are influenced by life stage and proximity to roads.

### Effects of life stage and roads on Bd‐inhibitory bacterial diversity and composition

**Fig. 3:**
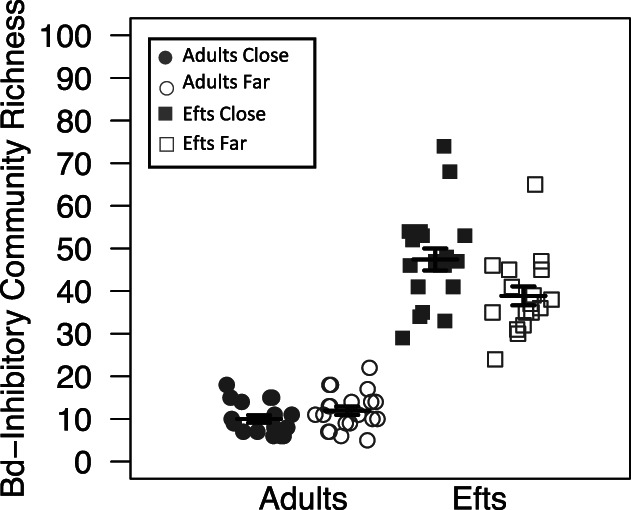
Community richness dot plot for Bd‐inhibitory data with mean (long horizontal line) and standard errors (short horizontal line). Each point represents the Bd‐inhibitory bacterial skin community of an individual sample; point colour indicates proximity to roads (grey is close to roads and white is far from roads) and shape identity indicates newt stage (circles are adult newts and squares are efts).

**Fig. 4:**
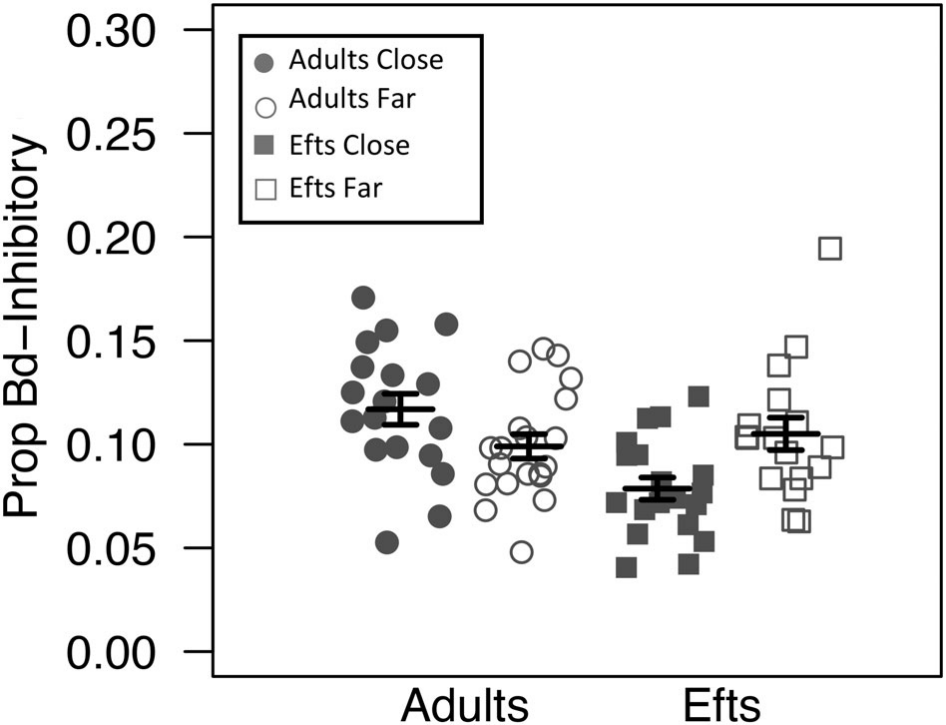
Community richness dot plot for the abundance of Bd‐inhibitory ASVs data with mean (long horizontal line) and standard errors (short horizontal line). Each point represents the Bd‐inhibitory bacterial skin community of an individual sample; point colour indicates proximity to roads (grey is close to roads and white is far from roads) and shape identity indicates newt stage (circles are adult newts and squares are efts).

We also evaluated whether differences in newt skin Bd‐inhibitory microbial richness and the proportion of Bd‐inhibitory bacteria, quantified as the number of Bd‐inhibitory bacteria reads divided by the rarefied sequence depth, exist between the two newt life stages (i.e. eft vs. adult) and populations that vary in their distance to roads. Using a backwards reduced fixed‐effect procedure to select the best fit linear mixed models, we identified a significant interaction between life stage and proximity to roads on putative Bd inhibitory community richness (*F*_1,74_ = 8.84, *p* = 0.004) and relative abundance (*F*_1,70.10_ = 23.74, *p* < 0.001). Adult newts (mean ± standard error: 11.03 ± 0.69) possessed less putative Bd inhibitory bacteria than efts (43.39 ± 1.84); however, the proportion of putative inhibitory bacteria reads was higher among the adult age class (adults: 0.51 ± 0.027; efts: 0.17 ± 0.0098). Efts living close to roads possessed the highest putative inhibitory bacteria richness (mean ± standard error: 47.42 ± 2.58), followed by efts living far from roads (38.88 ± 2.22), adults living far from roads (11.95 ± 0.99) and adults living close to roads (10.00 ± 0.90; Fig. 3). Among adults and efts, the proportion of putative Bd inhibitory bacteria was higher among individuals living far from roads (adults: 0.60 ± 0.03; efts: 0.20 ± 0.01) than conspecifics living close to roads (adults: 0.42 ± 0.03; efts: 0.15 ± 0.01; Fig. 4).

The indicator species analysis identified 11 Bd‐inhibitory ASVs associated with adults and were characterized as Comamonadaceae (eight ASVs), Sphingomonadaceae (two ASVs) and Oxalobacteraceae (one ASV; Table S2). Additionally, 91 Bd‐inhibitory ASVs were associated with efts. Notably, most of the ASVs associated with both groups of efts were identified to the phylum Proteobacteria and all had relative abundances of less than 1.2% of the total sample read count, indicating that the differences across life stages are based on mostly rare and soil‐associated putative Bd inhibitory ASVs (Table S2). Eight Bd‐inhibitory ASVs were associated with proximity to roads across all salamanders and were characterized as Pseudomonadaceae (two ASVs), Comamonadaceae (one ASV), Aeromonadaceae (one ASV), Rhizobiaceae (one ASV), Streptomycetaceae (one ASV), Microbacteriaceae (one ASV) and Xanthomonadaceae (one ASV). Finally, 8 Bd‐inhibitory ASVs were more abundant in populations living close to roads and were characterized as Comamonadaceae (three ASVs), Streptomycetaceae (two ASVs) and the remaining families of Sphingobacteriaceae, Burkholderiaceae and Sphingomonadaceae all represented by one ASV.

## Discussion

We demonstrated that life stage and proximity to roads influenced amphibian skin microbiotas. Specifically, we found that efts had higher overall richness and diversity compared to adults. The effect of proximity to roads on the skin microbiotas varied depending on life stage and the alpha diversity metric used. Compared to populations close to roadways, adults living far from roads exhibited higher Shannon diversity, while efts exhibited lower Shannon diversity. However, neither adults nor efts differed in phylogenetic diversity. Additionally, we found an effect of distance to roads on community composition, but life stage had the largest influence on turnover and community dispersion. The indicator species analysis identified that the effects of life stage and proximity to roads had the strongest effect on the turnover of rare ASVs. Next, we found that efts had higher Bd‐inhibitory richness compared to adults. In addition, efts living close to roads had higher overall and Bd‐inhibitory richness than those far from roads. However, when looking at the proportion of Bd‐inhibitory bacteria across all ASVs in our samples, we found that for adult newts and efts, individuals living close to roads had the higher proportion of Bd‐inhibitory bacteria compared to those living far from roads.

### Life stage strongly influences the skin microbiomes of eastern newts

Life stage had the largest impact on eastern newt skin microbiotas in our study. Our results are consistent with previous work demonstrating that shifts in development and habitat use (i.e. metamorphosis) can alter skin microbial diversity and community composition (Kueneman *et al*., 2014; Jimenez and Sommer, 2017). Indeed, we found that the relative abundances of microbes on aquatic versus terrestrial life stages were consistent with their respective habitats. The core microbiome of adult newts had higher relative abundances of the phyla Proteobacteria, GN02 and Verrucomicrobia, which are known to inhabit aquatic environments. In contrast, efts had higher relative abundances of Actinobacteria, Acidobacteria and Bacteroidetes in their core microbiotas, which are known to inhabit both terrestrial soil and newt microbiomes (Walke *et al*., 2014; Sabino‐Pinto *et al*., 2017; Buttimer *et al*., 2021). Additionally, efts in our study possessed microbiota richness and diversity that was approximately four times higher than that of adults. While we did not measure the environmental microbiotas in this study, the higher alpha diversities among efts could be related to eft interaction with terrestrial soils, which have been shown to possess more diverse microbiotas relative to other habitats (Thompson *et al*., 2017). In terms of life history, efts spend time in a residence phase and multiple migratory phases (Healy, 1975), and can move at an average of 30 m per night (Roe and Grayson, 2008), which may also allow them to pick up a more rich and diverse microbial community (Wolz *et al*., 2018).

While efts had higher overall diversity, adult newts had a higher proportion of putatively Bd‐inhibitory microbial diversity. Importantly, aquatic life stages are likely to have higher rates of exposure to fungal pathogens because moisture and temperature conditions associated with aquatic environments provide ideal conditions for fungal growth and spread (Berger *et al*., 1999). In 2013, a new fungal pathogen, *Batrachochytrium salamandrivorans* (Bsal), was found to cause mass die‐offs in European fire salamanders (*Salamandra salamandra*; Martel *et al*., 2013). Studies suggest that Bsal is highly pathogenic to other salamanders, including newts (Martel *et al*., 2014). While Bsal is not currently found in North America, it has the potential to spread and devastate populations across North America (Yap *et al*., 2015; Yap *et al*., 2017). In this study, we focused on Bd‐inhibitory bacteria as a proxy for potential responses to Bsal because it has been demonstrated that Bd‐inhibitory bacteria can also inhibit the growth of Bsal (Muletz‐Wolz *et al*., 2017; Woodhams *et al*., 2018). As emerging diseases threaten salamanders worldwide, studies that continue to explore the potential for risk‐matching shifts in microbiota across life stages have critical conservation implications. Therefore, understanding the link between Bd‐inhibitory bacteria, host life history and anthropogenic activities may provide vital information for conserving salamander populations in the future.

### The effect of proximity to roads on eastern newt skin microbiomes depends on life stage

The effect of roads on the skin microbiota of eastern newts varied with life stage and community diversity metric. Efts living close to roads possessed higher bacterial richness than those far from roads. In contrast, adults living far from roads exhibited higher Shannon diversity than efts. These patterns suggest that eft skin microbiotas respond to road disturbance through a gain/loss of microbial species, but adults experience changes in the distribution of species abundances. While we cannot pinpoint the mechanism behind the interaction of age class and proximity to roads, our observations likely result from road‐induced changes to abiotic and/or biotic factors. For example, in both aquatic and terrestrial habitats, changes in salt and nitrogen pollutant concentrations associated with roads can lead to turnover in environmental community assemblies of microbes in the environment (Fornier *et al*., 2021). For example, recent work demonstrates that road salts can impose a strong selective pressure on soil microbiotas, leading to increased frequencies of halotolerant bacteria in road‐proximate soils (Bowen *et al*., 2021). While most environmental microbiological work has focused on evaluating the effect of roads on free‐living microbiomes, to our knowledge, ours is the first study to demonstrate that proximity to roads and life stage interactively alter an animal's microbiome.

The interconnect between habitat quality, host‐associated microbial diversity/function and infectious disease resistance highlights the need to integrate techniques from microbial ecology into amphibian disease and conservation research. Using the indicator species analysis, we were able to identify abundant ASVs associated with life stage and proximity to roads. The analysis on the overall bacteria assigned numerous ASVs to each category, but only one ASV in the genus *Pedobacter*, which is a known Bd‐inhibitory bacterium (Harris *et al*., 2006), was found at a relative abundance greater than 1%. All remaining ASVs that were assigned to the other categories were mostly rare. This suggests that the environment may have had a disproportionate effect on the persistence of distinct rare bacteria in newts. Additionally, we found high relative abundances of Bd‐inhibitory bacteria from the family Comamonadaceae in adult eastern newts close (58.67% relative abundance) and far (39.94% relative abundance) from roads. This family includes many bacteria that are known to live in aquatic environments (Willems, 2014). For example, Sabino‐Pinto *et al*. (2017) also found Comamonadaceae at high relative abundance in the adult, aquatic form of marbled newts (*Triturus marmoratus*). Specifically, within the family Comamonadaceae, we found that the genus *Hydrogenophaga* was common in adults close and far from roads. *Hydrogenophaga* are ubiquitous free‐living freshwater planktonic bacteria (Willems *et al*., 1989). Importantly, this bacterium has been previously categorized as being part of the core microbiome of three newt species in Colorado, and is also common across other amphibian species, fish and zooplankton (Grossart *et al*., 2009; Walke *et al*., 2014; Bates *et al*., 2019). In the efts, we found the most abundant bacteria were from the families Pseudomonadaceae (close = 1.94% average relative abundance; far = 4.04%) and Xanthomonadaceae (close = 1.64%; far = 2.24%). Interestingly, these two bacterial families have been noted as being highly abundant in four other newt species (family Salamandridae) in aquatic environments (Bletz *et al*., 2017). The family Pseudomonadaceae is very functionally diverse group that includes many commensals, pathogens and beneficial bacteria to vertebrate hosts. Pseudomonads are a potentially important group of salamander symbionts, as it includes isolates previously collected from amphibians with the ability to produce defensive toxins (i.e. tetrodotoxin) and antifungal metabolites (Harris *et al*., 2006; Vaelli *et al*., 2020). In our observations, proximity to roads had a negative effect on the relative abundance of these families and potentially a consequential reduction in their potential protective functions. We acknowledge that using a database to identify the function of these bacteria limits our ability to accurately tie ecological functions with ASVs. Therefore, it is critical for future studies to evaluate the functionality of specific bacteria against pathogens using traditional microbiological methods or metagenomics.

### Conclusions and future directions

Host microbial diversity and composition is highly influenced by life stage and habitat, but can also respond to anthropogenic habitat modification associated with proximity to roads. Importantly, the effects of these natural and anthropogenic changes are interactive. While our study identified abundant ASVs within these populations, characterizing the functional diversity of the microbiota will be imperative for understanding the mechanisms behind the microbial changes observed. Notably, amphibians worldwide are declining due to habitat modification and emerging infectious diseases. Specifically, newt populations are currently threatened by the emerging infectious disease *B*. *salamandrivorans*. Given the link between host‐associated microbiotas and host health, it is imperative that future studies continue to integrate microbial ecology techniques. For amphibians, the integration of these two fields may help not only identify potential threats to hosts (e.g. pathogens and pollutants) but could lead to insights on how to successfully develop and manage life‐stage specific natural populations. As we continue to gain insight into the natural and anthropogenic effects on the amphibian microbiota, it may be useful for researchers to construct a model to link these effects to amphibian host health (e.g. fitness and population growth). Ultimately, understanding community‐level interactions from the host‐associated microbes up to the environment could help to contribute to the development of more effective conservation strategies that allow managers to deploy cost‐effective conservation efforts that identify and target natural populations that are most at risk.

## Experimental procedures

### Field methodology

We sampled newts in two populations located far (>250 m) from roads in rural areas (AT and FS), and two located close (<250 m) to roads in urban areas (NP and NH; Table 1). All sites consisted of a pond surrounded by forested habitat. The side distances from roads were measured from the centre of the collection site to the nearest road using Google Earth Pro. Adult newts were collected from a pond at each location using a dip net. Once an adult newt was found, it was placed in a new, plastic bag with ~100 ml of pond water. Terrestrial efts were collected by hand through visual encounter surveys in forested habitat within 100 m of the pond at each site. Once an eft was found, it was placed in a new, plastic bag. All newts were handled with gloves, and new gloves were donned between the handling of each newt. All newts from a site were collected within 1 h of each other and were brought back to the laboratory for processing. We rinsed each newt with 250 ml of sterile, nanopure water and swabbed the dorsal and ventral surface separately with sterile cotton swabs 30 times for skin microbiota collection. We stored the dorsum swabs in a sterile 1.5‐ml microcentrifuge tube and stored them at −80°C. The ventral surface swabs were used for bacterial culturing in a companion study. All newts were returned to their location of capture immediately after sampling. Upon completion of the experiment, the skin swab samples were shipped overnight to the University of California, Berkeley on dry ice. Upon receipt, the samples were stored at −80°C until DNA extraction.

### DNA extraction, PCR amplification and sequencing

We isolated DNA from dorsum skin swab samples using the DNeasy PowerSoil DNA Isolation Kit (Qiagen N.V., Hilden, Germany) following the modifications to the manufacturer's protocol described in Hernández‐Gómez *et al*. (2017b). To control for contamination, we included unused swabs (i.e. negative extraction controls) and researcher glove swabs in our DNA extractions. We amplified the bacterial 16S rRNA V4 region using primer pair F515/R806 with the attachment of connector sequences that allows for the attachment of barcode/sequencing adaptors (Hernández‐Gómez *et al*., 2017a). We ran each sample in triplicate, and each reaction consisted of 5.0 μl of template DNA, 7.5 μl of 2× MyTaq Master Mix (Bioline, Taunton, MA, USA), 1.0 μl of 1 nM forward and reverse primers and 1.5 μl of sterile water for a total of 15 μl per reaction. PCR conditions consisted of 94°C for 3 min, 30 cycles of 94°C for 45 s, 50°C for 60 s and 72°C for 90 s, followed by 72°C for 10 min. We pooled amplicon triplicates and cleaned the products using the UltraClean PCR Clean‐up kit (Qiagen N.V.).

We performed a second PCR on microbiota amplicons to ligate dual‐index barcodes paired with Illumina sequencing adaptors (Hernández‐Gómez *et al*., 2017a) to the ends of amplicons. The PCR consisted of 5.0 μl of clean amplicons, 7.5 μl 2× MyTaq Master Mix, 1.0 μl of 1 nM forward and reverse barcode primers, and 1.5 μl of water for a total of 15 μl reactions. PCR conditions consisted of 94°C for 3 min, 5 cycles of 94°C for 45 s, 65°C for 60 s and 72°C for 90 s, followed by 72°C for 10 min. We quantified the PCR products using a Qubit Fluorometer (Invitrogen Corp., Carlsbad, CA, USA), pooled samples in equimolar amounts, and cleaned the sample pool using the UltraClean PCR Clean‐Up kit. We overnighted the barcoded sample pool on dry ice to the Cornell University Biotechnology Resource Centre. The sample pool was sequenced on a MiSeq machine (Illumina, San Diego, CA, USA) using the reagent kit V2 to produce 250‐bp paired‐end reads.

### Microbiota sequence analysis

We processed raw sequencing reads using Trimmomatic (Bolger *et al*., 2014) to remove adapter sequences, bases below threshold quality of phred‐20 from both ends of reads, and any resulting reads under 30 bp. We paired reads that passed initial quality control using PANDAseq (Masella *et al*., 2012). Only reads that paired successfully were employed in subsequent analysis.

Our microbiota sequence analysis consisted of established sequence read processing pipelines to filter erroneous reads, generate an ASV (error‐corrected unique DNA sequences) table, create a representative sequence phylogeny and assign taxonomy to ASVs. We chose to use ASVs rather than operational taxonomic units, because ASVs provide greater resolution in amplicon differentiation (Callahan *et al*., 2017). We processed the resulting read file using the Quantitative Insights into Microbial Ecology version 2.2020.2 (QIIME2) pipeline (Bolyen *et al*., 2019). We processed reads using the DADA2 plugin to quality filter, dereplicate, remove chimaeras and denoise reads using default settings (Callahan *et al*., 2016). We aligned ASV representative sequences using MAFFT and generated a phylogenetic tree in FastTree2 to be used in alpha and beta analyses (Price *et al*., 2010; Katoh and Standley, 2013). We applied a pre‐trained Naïve Bayes classifier on the Greengenes 13_8 database to assign taxonomy to each ASV at the genus level (DeSantis *et al*., 2006). We ran the ASV table through the package decontam in R to identify ASVs associated with negative extraction controls and removed identified contaminants from the ASV table (Davis *et al*., 2018). In addition, we filtered out any ASVs whose taxonomy matched chloroplast or mitochondria as these were not the target of our amplification protocol. To standardize sequencing depth throughout all samples, we rarefied the filtered ASV table to 5991 sequences per sample.

### Statistical analysis

We transferred the rarefied ASV table and Newick phylogeny to R (version 3.5.1). A Shapiro–Wilk test in R was implemented on all univariate dependent variables to evaluate normality prior to statistical model selection. We calculated three distinct alpha diversity metrics per individual using the R packages *vegan* and *picante*: community richness (i.e. number of ASVs observed per sample), evenness (i.e. Shannon diversity indices) and phylogenetic diversity (i.e. Faith's phylogenetic diversity; Oksanen *et al*., 2007; Kembel *et al*., 2010). To assess differences in community composition across samples, we applied the R packages *GuniFrac* and *vegan* to calculate pairwise unweighted and weighted UniFrac beta diversity metrics (Chen *et al*., 1995; Oksanen *et al*., 2007). We chose to include these beta diversity metrics as they account for differences in presence/absence of phylogenetic lineages among samples (unweighted UniFrac) and abundance‐based differences in phylogenetic lineages among samples (weighted UniFrac). We used the package *vegan* to calculate the beta diversity dispersion or distances of samples to the centroid of their corresponding group (e.g. adults living close to roads, adults living far from roads, efts living close to roads and efts living far from roads) for both unweighted and weighted UniFrac distances. Finally, we calculated the core microbiome for each group of salamanders using the package *microbiome* by identifying ASVs that were present in more than 50% of individuals (Lahti and Shetty, 2018).

#### Effects of life stage and roads on bacterial diversity and composition

To evaluate differences in alpha diversity between newt samples, we used community richness, Shannon diversities and phylogenetic diversities as dependent variables and population as a random effect in linear mixed‐effect models. We tested differences in alpha diversity based on newt stage, distance to roads, and the interactive effect of newt stage and distance to roads. We used a backwards reduced fixed‐effect procedure to exclude non‐significant variables [R package *lmerTest*; (Kuznetsova *et al*., 2015]. The significance of the final models was assessed with likelihood ratio tests (LRTs). We ran a post hoc univariate analysis of variance (ANOVA) with Bonferroni correction to determine if the differences in alpha diversities were significant between newt stage and proximity to roads (SPSS 25). To characterize the effect of life stage, distance to roads and their interaction on the community composition and structure of newt skin microbiota, we conducted two PERMANOVA tests (R package *vegan*) using weighted UniFrac and unweighted UniFrac matrices as dependent variables and life stage and distance identity as a random variable. We produced NMDS plots using the two dissimilarity matrices to visualize clustering of samples by life stage and distance to roads. We also implemented linear mixed models with backwards reduction to assess how unweighted and weighted UniFrac dispersion (dependent variables) varied with proximity to roads (fixed variable), life stage (fixed variable), the interaction between road proximity and life stage (fixed variable) and population (random variable). Finally, we implemented an indicator species analysis using the R package *indicspecies* to identify ASVs whose relative abundance differs between skin samples collected from eft and adult newts, along with distance to roads (De Caceres *et al*., 2016).

#### Effects of life stage and roads on Bd‐inhibitory bacterial diversity

We used Bd‐inhibitory microbial diversity and proportion of overall richness as dependent variables and population as a random effect in linear mixed‐effect models (R package *lmerTest*). We used a backwards reduced fixed‐effect procedure to pick out non‐significant variables. The significance of both final models was calculated with LRTs. We ran a *post hoc* univariate ANOVA with Bonferroni correction to determine if the differences in Bd‐inhibitory community richness and abundance were significant between life stage and proximity to roads (SPSS 25). Finally, we implemented an indicator species analysis using the R package *indicspecies* to identify Bd‐inhibitory ASVs whose relative abundance differs between skin samples collected from eft and adult newts, along with distance to roads.

## Supporting information


**Table S1.** Amplicon Sequence Variants (ASVs) among adults and efts close and far from roads. The ASV list is broken down by ASV associations to sample group(s). The average relative abundance of each ASV is displayed for each treatment group (Adults Close; Adults Far; Efts Close; Efts Far). The relative abundance cells are shaded based on their value with 0 in white and the max value in black. For each ASV, the association statistic and corresponding p value are presented as well as its taxonomy.

**Table S2.** Bd‐inhibitory Amplicon Sequence Variants (ASVs) among adults and efts close and far from roads. The ASV list is broken down by ASV associations to sample group(s). The average relative abundance of each ASV is displayed for each treatment group (Adults Close; Adults Far; Efts Close; Efts Far). The relative abundance cells are shaded based on their value with 0 in white and the max value in black. For each ASV, the association statistic and corresponding p value are presented as well as its taxonomy.
