# Supplementary material for: Life stage and proximity to roads shape the skin microbiota of eastern newts (Notophthalmus viridescens)
Source: Environ Microbiol. 2022 Apr 29;24(9):3954–65. doi: 10.1111/1462-2920.15986 (PMC9790580; doi:10.1111/1462-2920.15986)
Supplement: Supplementary file 2 — Table S2. Bd‐inhibitory Amplicon Sequence Variants (ASVs) among adults and efts close and far from roads. The ASV list is broken down by ASV associations to sample group(s). The average relative abundance of each ASV is displayed for each treatment group (Adults Close; Adults Far; Efts Close; Efts Far). The relative abundance cells are shaded based on their value with 0 in white and the max value in black. For each ASV, the association statistic and corresponding p value are presented as well as its taxonomy. [file EMI-24-3954-s002.docx]

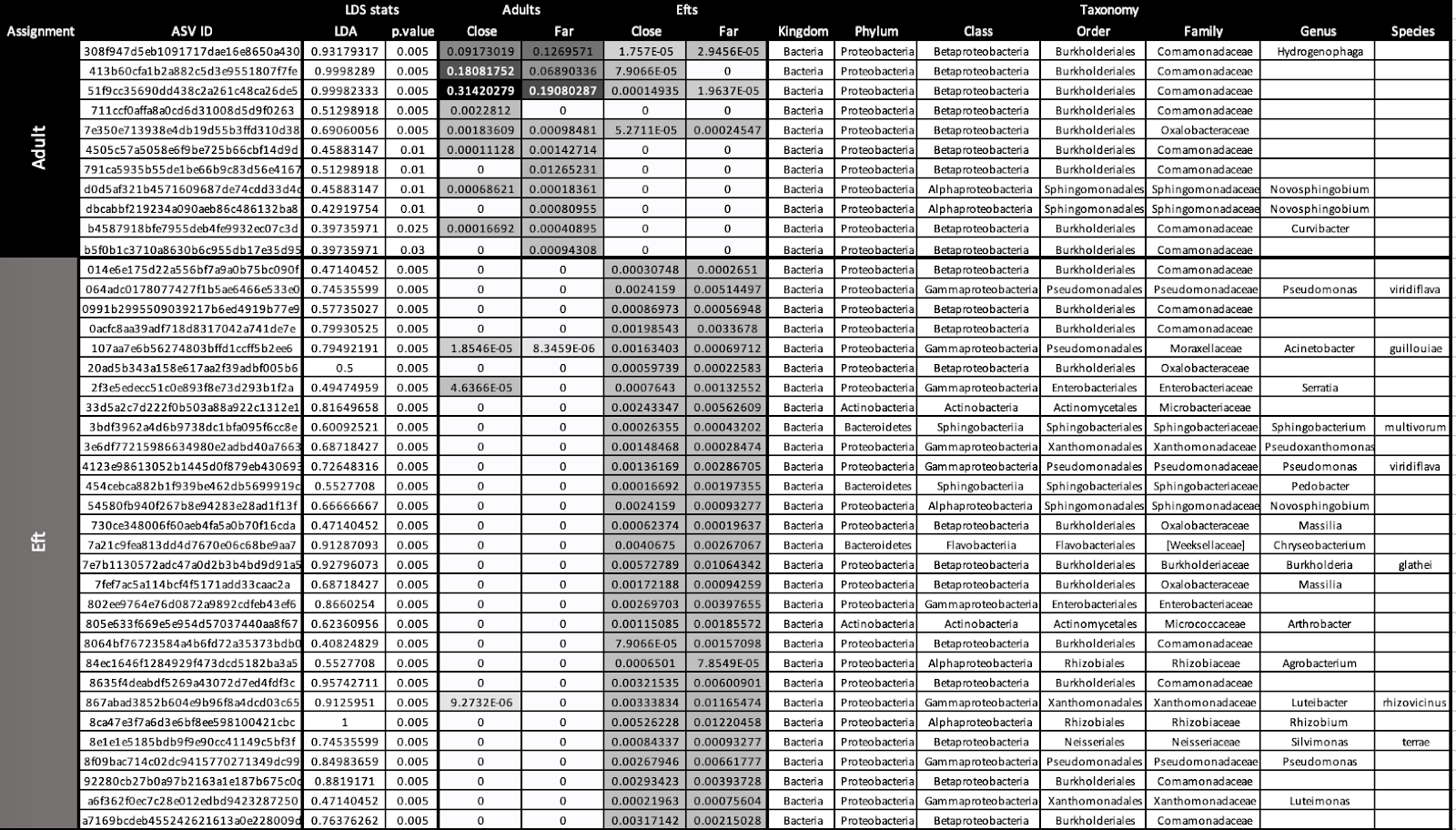


**Table S2.** Bd-inhibitory Amplicon Sequence Variants (ASVs) among adults and efts close and far from roads. The ASV list is broken down by ASV associations to sample group(s). The average relative abundance of each ASV is displayed for each treatment group (Adults Close; Adults Far; Efts Close; Efts Far). The relative abundance cells are shaded based on their value with 0 in white and the max value in black. For each ASV, the association statistic and corresponding p value are presented as well as its taxonomy.


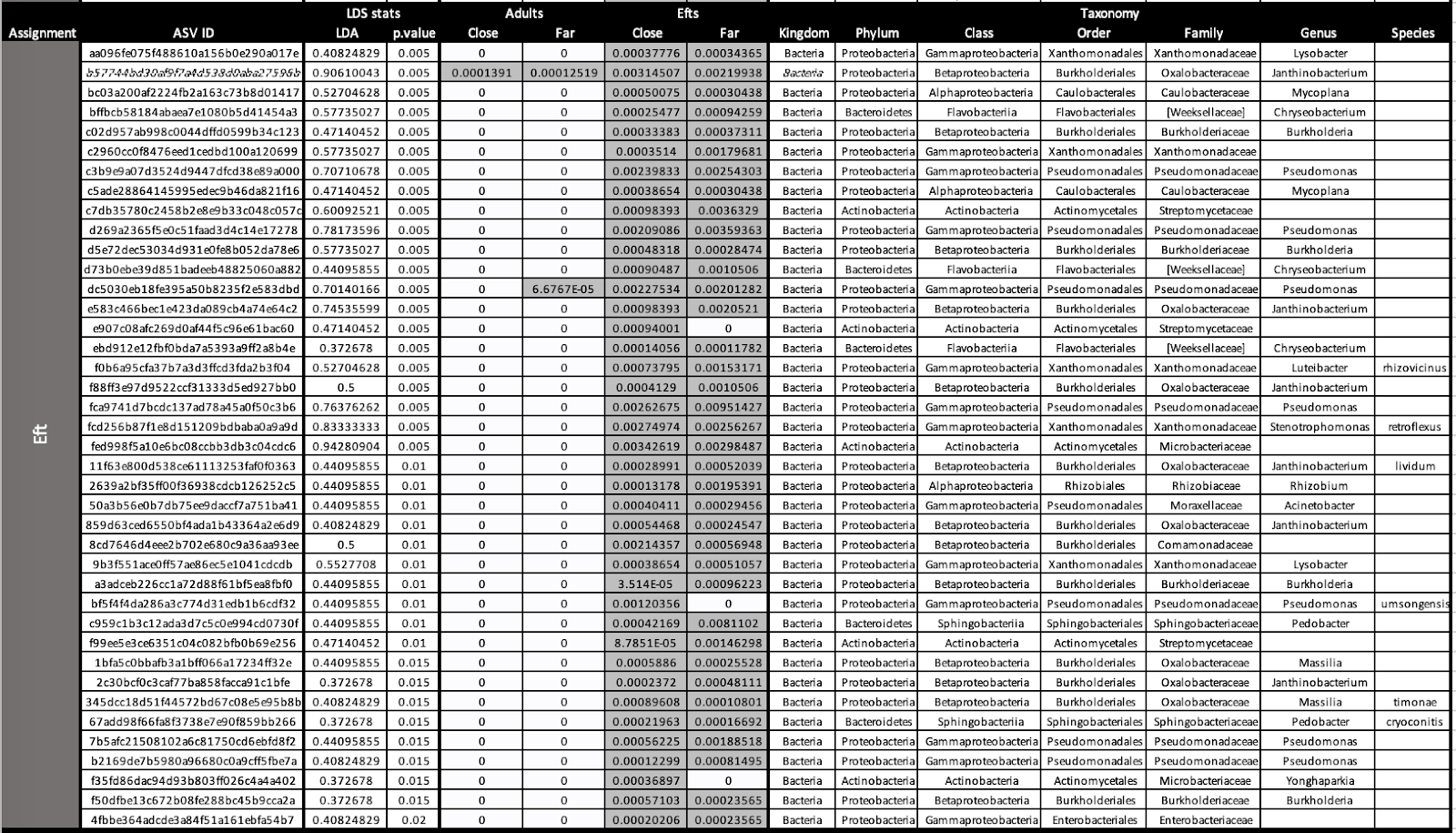


**Table S2.** Continued.


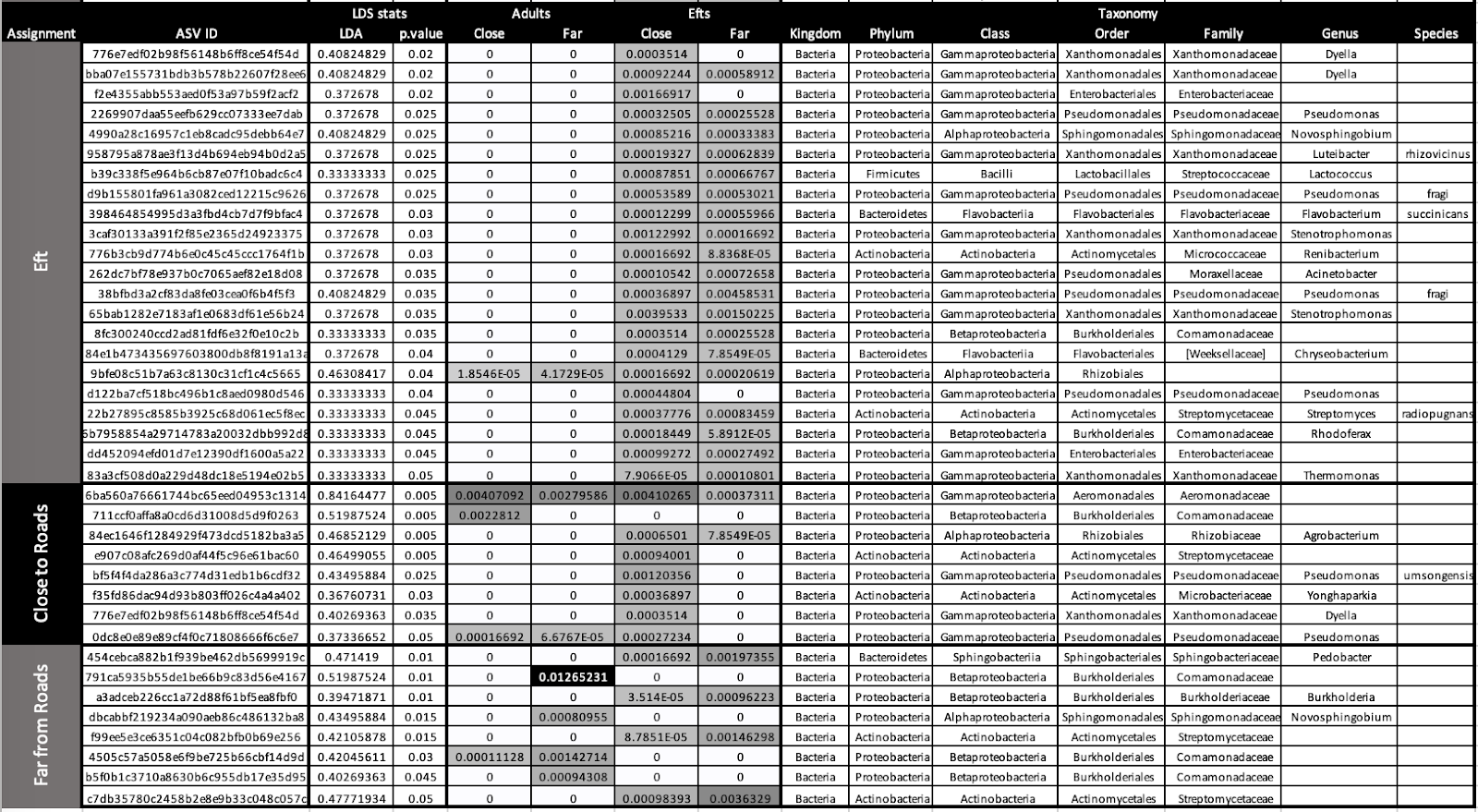


**Table S2.** Continued.
